# Supplementary material for: A quantitative account of mammalian rod phototransduction with PDE6 dimeric activation: responses to bright flashes
Source: Open Biol. 2020 Jan 8;10(1):190241. doi: 10.1098/rsob.190241 (PMC7014685; doi:10.1098/rsob.190241)
Supplement: Bright-flash responses from literature [file rsob190241supp1.pdf]

Open Biology, http://dx.doi.org/10.1098/rsob.19.0241

Supplementary Material

A quantitative account of mammalian rod phototransduction with PDE6 dimeric activation: Responses to bright flashes

Trevor D. Lamb[1] and Timothy W. Kraft[2]

[1]Eccles Institute of Neuroscience, John Curtin School of Medical Research, The Australian National University, ACT 2600, Australia, and [2]Department of Optometry and Vision Science, University of Alabama at Birmingham, Birmingham, AL, USA.

Supplementary Figure S1: Legend

Examples from the literature of bright-flash electrical response families recorded from mammalian rod photoreceptors using the suction pipette method.

Each panel shows a published family of responses to flashes of increasing intensity presented to a dark-adapted mammalian rod photoreceptor, and in each case an upward deflection represents a reduction in circulating current. The dark current level is indicated by the baseline before time zero, and (except in panel D) the zero current level is apparent as the peak level of the brightest response. Flash duration was typically 10–20 ms; wavelength was ~500 nm; flash intensity typically increased by a factor of 2–4 between traces. The estimated intensities of the dimmest and brightest flashes in each panel are shown in R*/rod near the traces; the rod's collecting area was taken to be 0.5 – 1 μm^2, in line with the authors' data.

A. Monkey rod from Fig. 1 of Baylor, Nunn & Schnapf (1984) [1]. *Macaca fascicularis*. Locke solution, bicarbonate buffer, 36 °C. 500 nm, 11 ms flashes. Intensities: 1.7 – 503 photon μm^{-2}; collecting area ~1 μm^2.

B. Rabbit rod from Fig. 1 of Nakatani, Tamura & Yau (1991) [2]. Locke solution, 41 °C. 500 nm, 8 ms flashes. Intensities: 7.6, 14, 28, 55, 110, 210, 410 and 1600 photon μm^{-2}; collecting area ~0.5 μm^2.

C. Human rod from Fig. 1 of Kraft, Schneeweis & Schnapf (1993) [3]. Locke solution, 37 °C. 500 nm, 10 ms flashes, in increments of ~2×, from 7–3060 photon μm^{-2}; collecting area ~1 μm^2.

D. Monkey rod from Fig. 2 of Kraft & Schnapf (1998) [4]. *Macaca fascicularis*. Locke solution, 37 °C. 500 nm. Flash intensities: 350, 630 and 1260 photon μm^{-2}; trials averaged: 54, 46 and 9, respectively; collecting area ~1 μm^2. Maximal response, 25 pA. This panel is included to show recovery of the mean response at very late times.

E. Mouse rod from Fig. 4A of Burns & Pugh (2010) [5]. Sv129 WT mouse. 10 ms flashes, in intensity increments of 2–4×, from 8 to 86,000 photon μm^{-2}.

F. Mouse rod from Sakurai et al (2011) [6]. C57BL6 WT mouse. Locke solution, 34–37 °C. 500 nm, 20 ms flashes, in increments of 0.5 $\log_{10}$ units from a dimmest of 3.9 photon μm^{-2}; collecting area ~0.5 μm^2.

Supplementary Figure S1

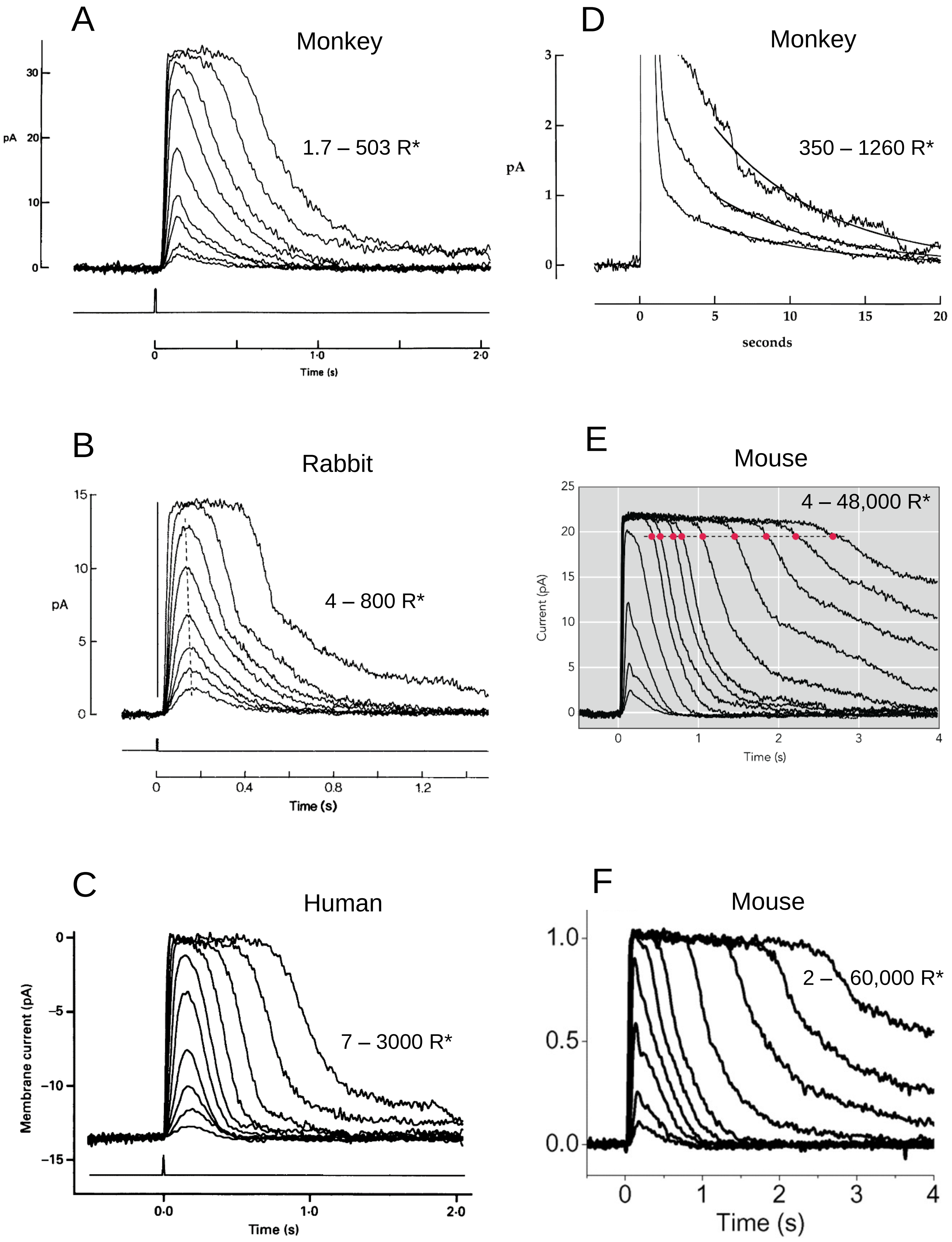

References

1. Baylor DA, Nunn BJ, Schnapf JL. 1984 The photocurrent, noise and spectral sensitivity of rods of the monkey Macaca fascicularis. *J. Physiol.* **357**, 575–607.

2. Nakatani K, Tamura T, Yau KW. 1991 Light adaptation in retinal rods of the rabbit and two other nonprimate mammals. *J. Gen. Physiol.* **97**, 413–435. (doi:10.1085/jgp.97.3.413)

3. Kraft TW, Schneeweis DM, Schnapf JL. 1993 Visual transduction in human rod photoreceptors. *J. Physiol.* **464**, 747–765.

4. Kraft TW, Schnapf JL. 1998 Aberrant photon responses in rods of the macaque monkey. *Vis. Neurosci.* **15**, 153–159.

5. Burns ME, Pugh EN. 2010 Lessons from photoreceptors: turning off G-protein signaling in living cells. *Physiology* **25**, 72–84. (doi:10.1152/physiol.00001.2010)

6. Sakurai K, Young JE, Kefalov VJ, Khani SC. 2011 Variation in rhodopsin kinase expression alters the dim flash response shut off and the light adaptation in rod photoreceptors. *Invest. Ophthalmol. Vis. Sci.* **52**, 6793–6800. (doi:10.1167/iovs.11-7158)
