# Supplementary figures and images for: A quantitative account of mammalian rod phototransduction with PDE6 dimeric activation: responses to bright flashes
Source: Open Biol. 2020 Jan 8;10(1):190241. doi: 10.1098/rsob.190241 (PMC7014685; doi:10.1098/rsob.190241)

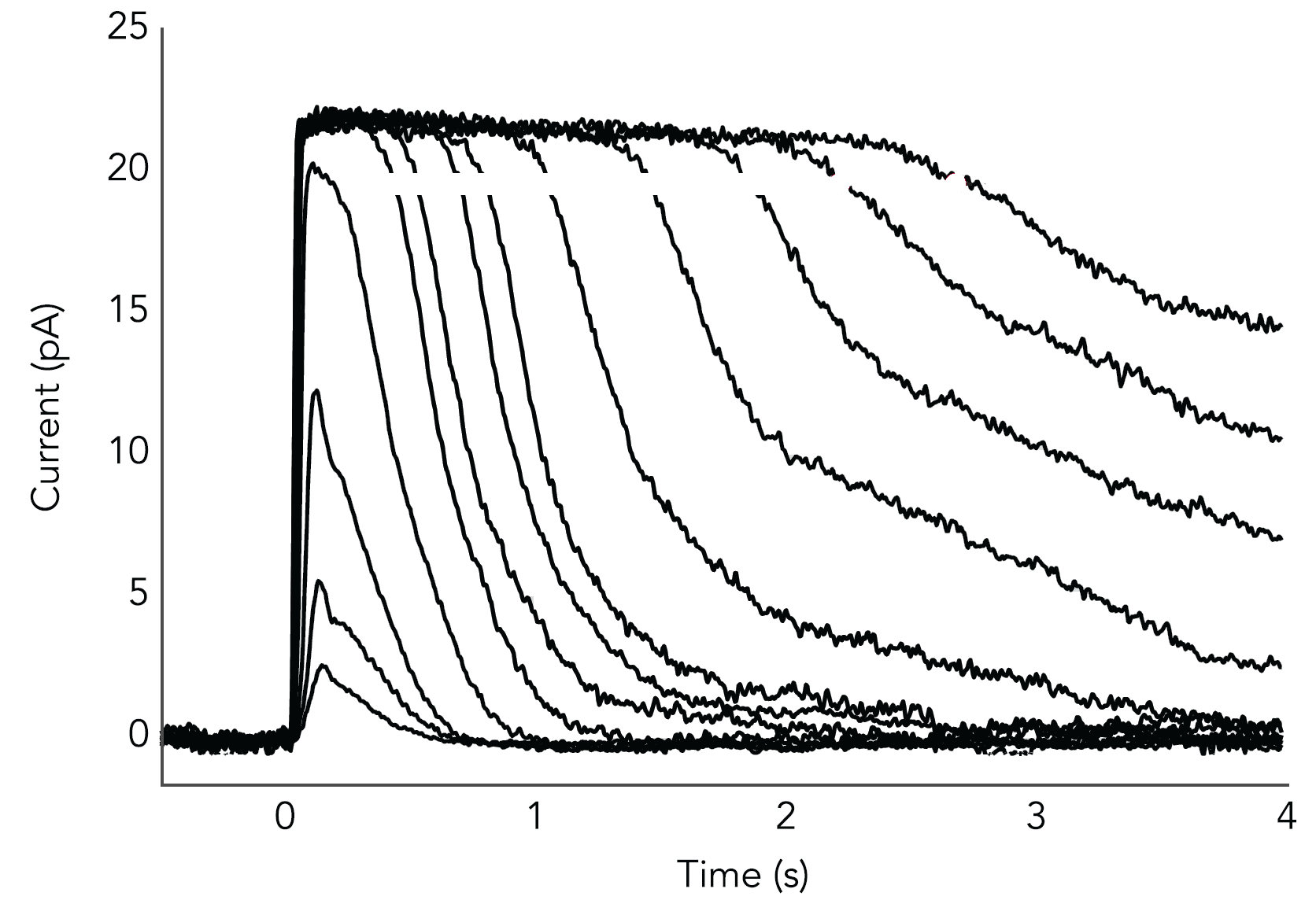

Supplement: Code and simulated data [file rsob190241supp2.zip › Data/Burns & Pugh (2010) Fig. 4A - Mouse - Unboxed.png]

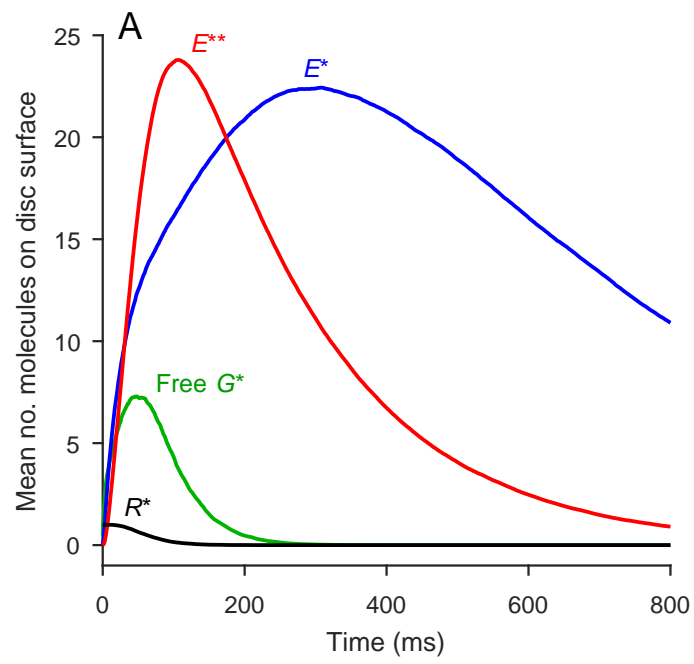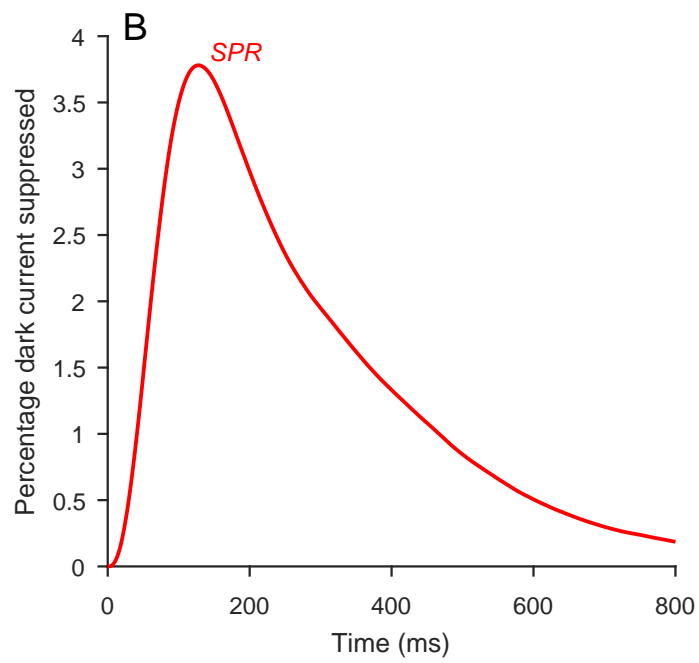

Supplement: Code and simulated data [file rsob190241supp2.zip › Figures/Fig_02_SPR.pdf]

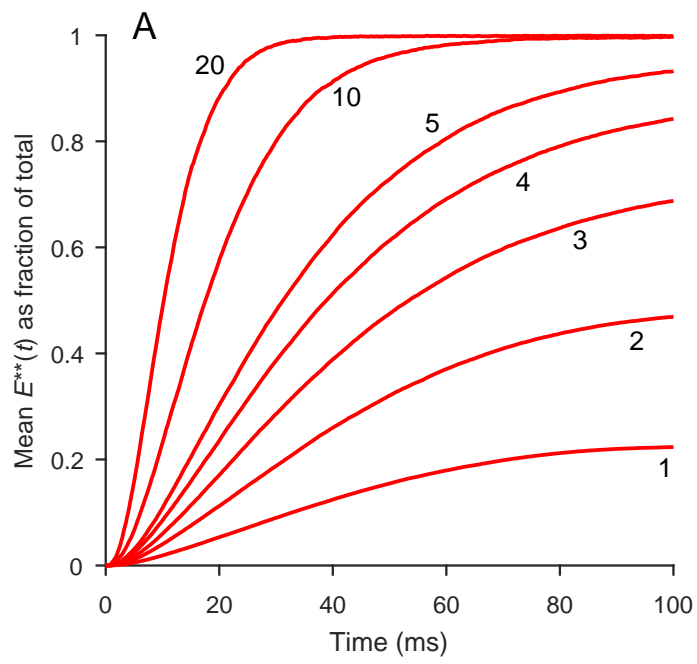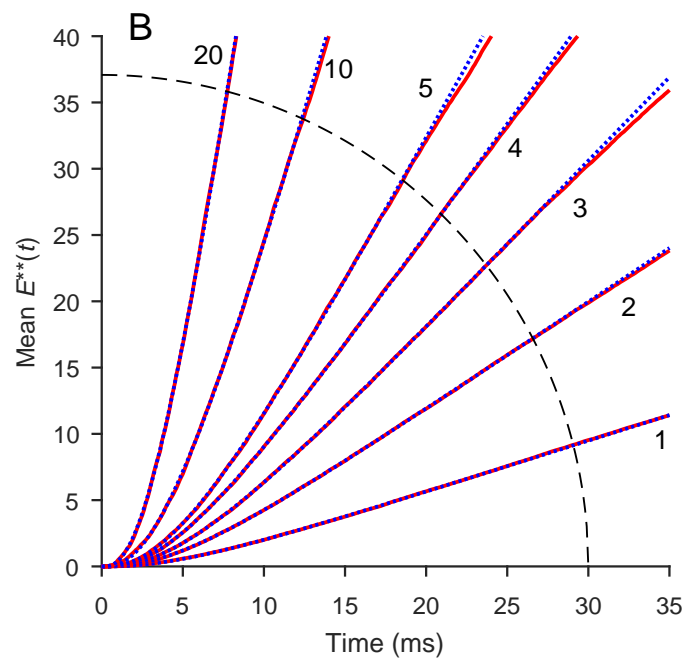

Supplement: Code and simulated data [file rsob190241supp2.zip › Figures/Fig_03_Rising.pdf]

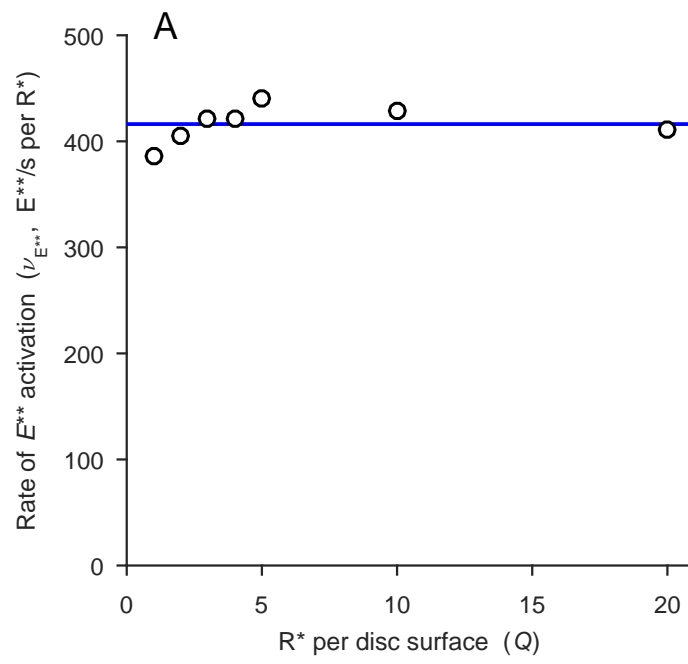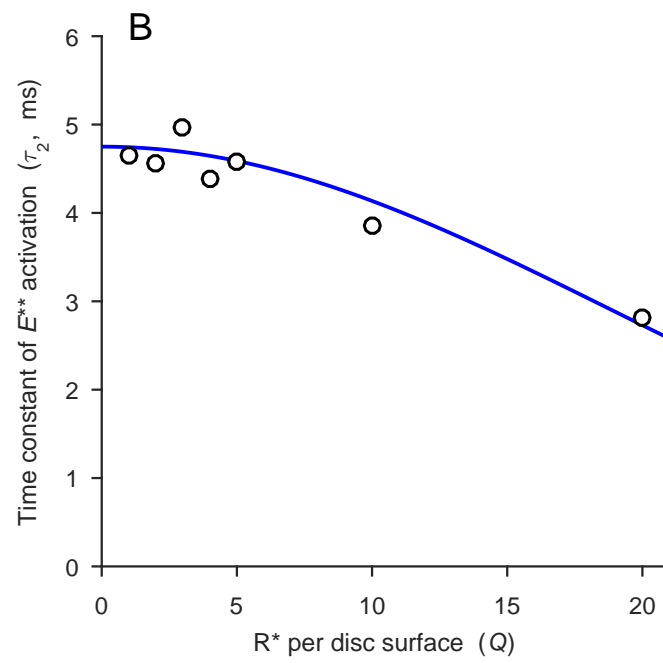

Supplement: Code and simulated data [file rsob190241supp2.zip › Figures/Fig_04_Rising_parameters.pdf]

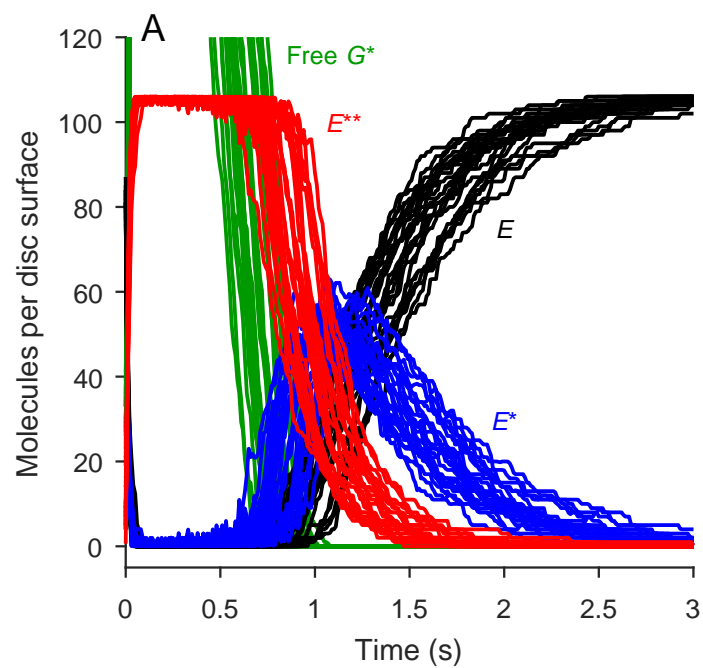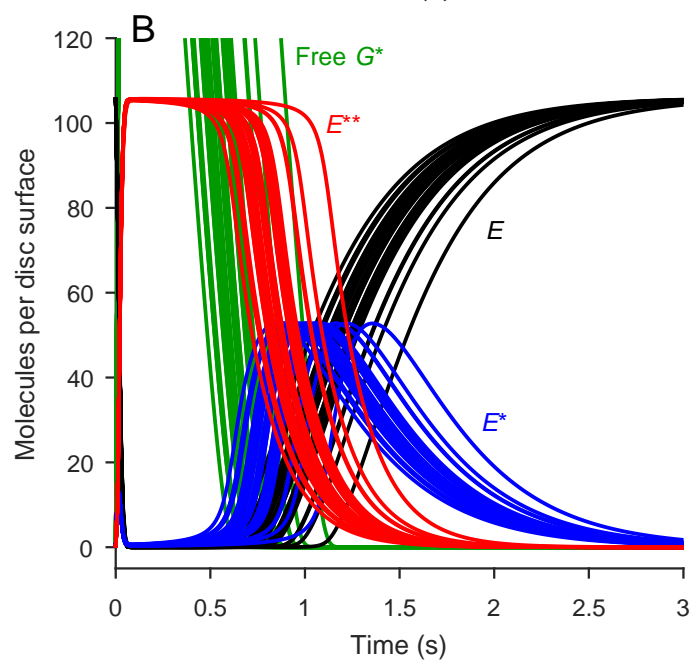

Supplement: Code and simulated data [file rsob190241supp2.zip › Figures/Fig_05_Recovery_Samples.pdf]

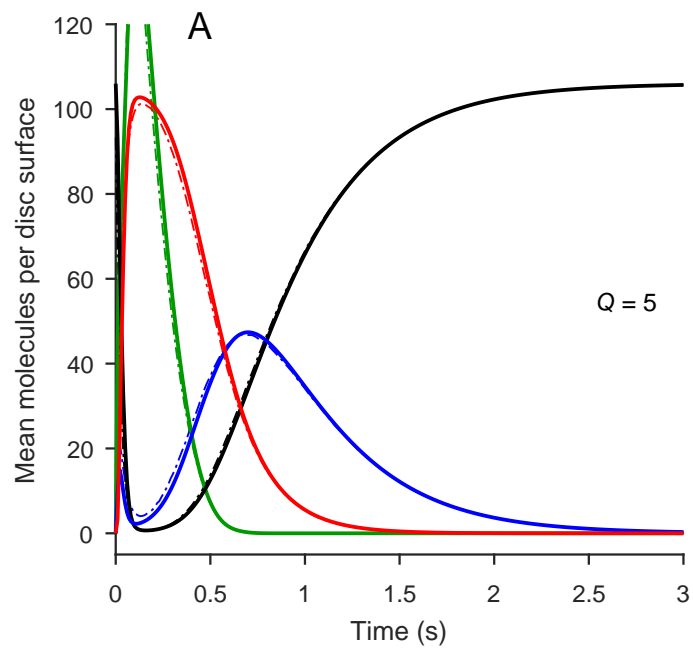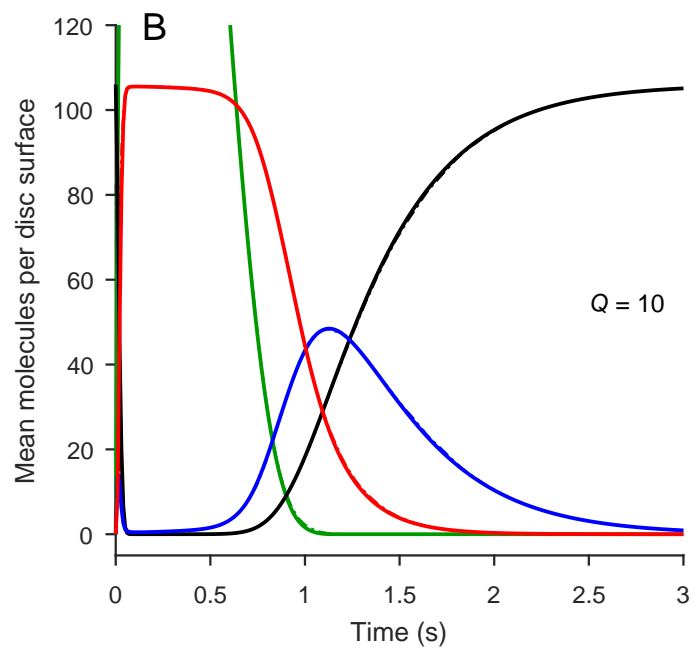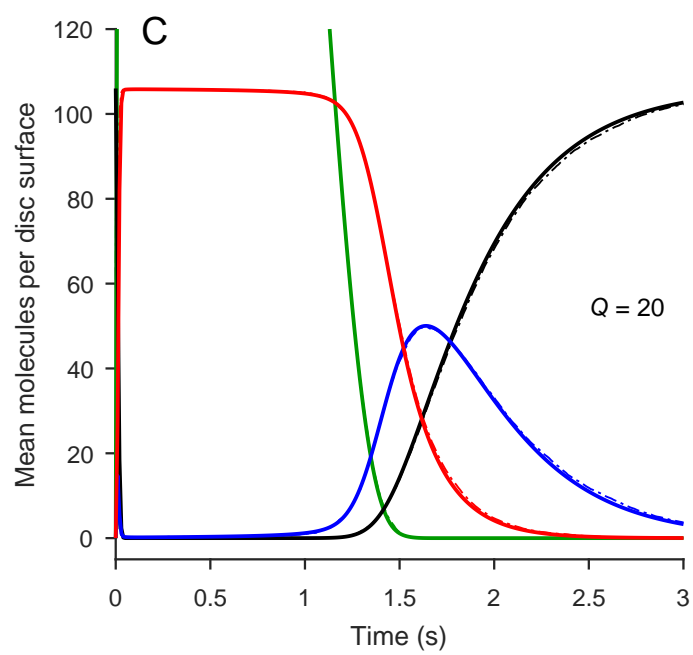

Supplement: Code and simulated data [file rsob190241supp2.zip › Figures/Fig_06_Recovery_Means.pdf]

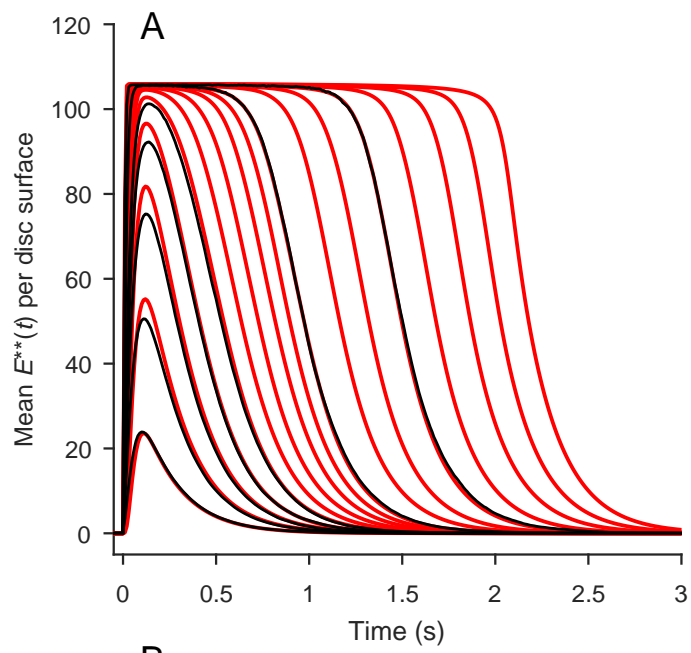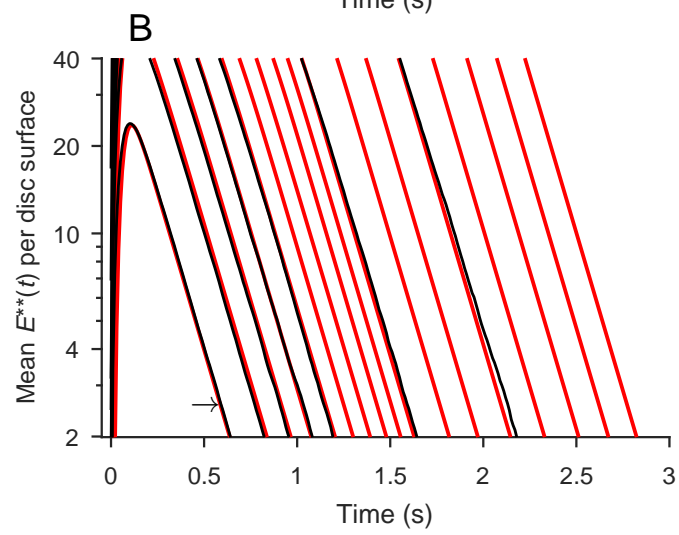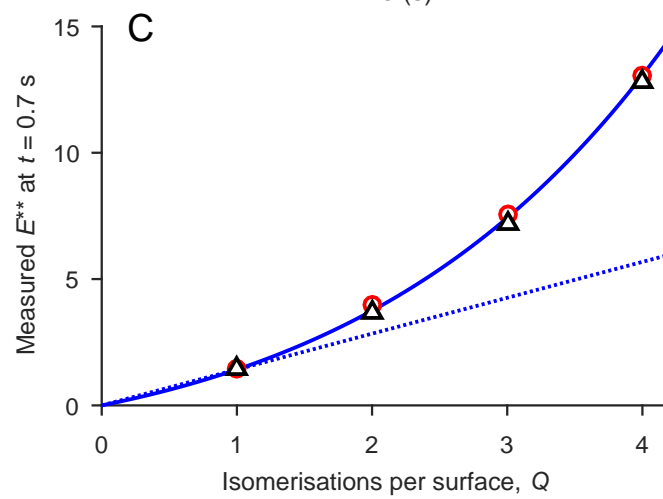

Supplement: Code and simulated data [file rsob190241supp2.zip › Figures/Fig_07_Recovery_Collected.pdf]

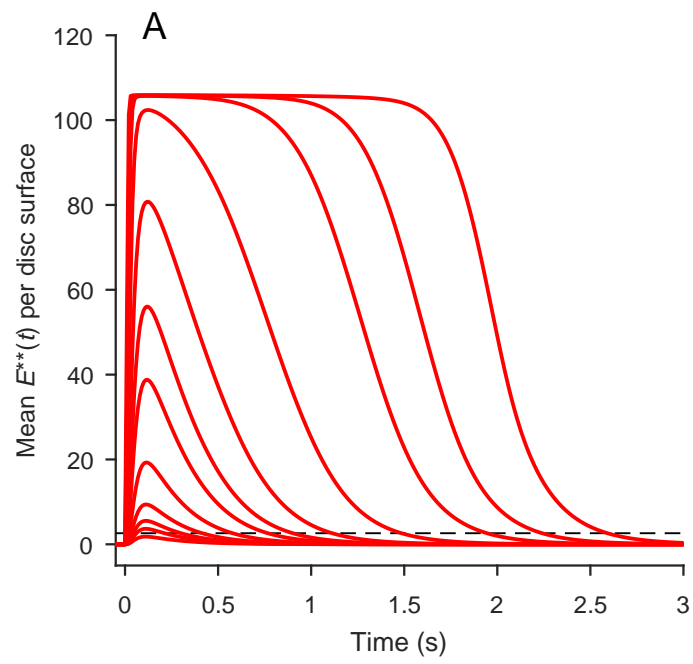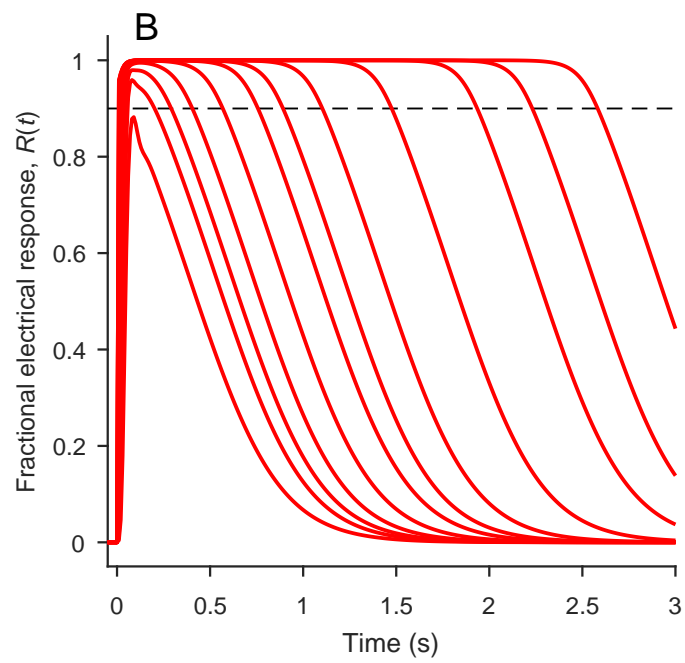

Supplement: Code and simulated data [file rsob190241supp2.zip › Figures/Fig_08_Recovery_v_Phi.pdf]

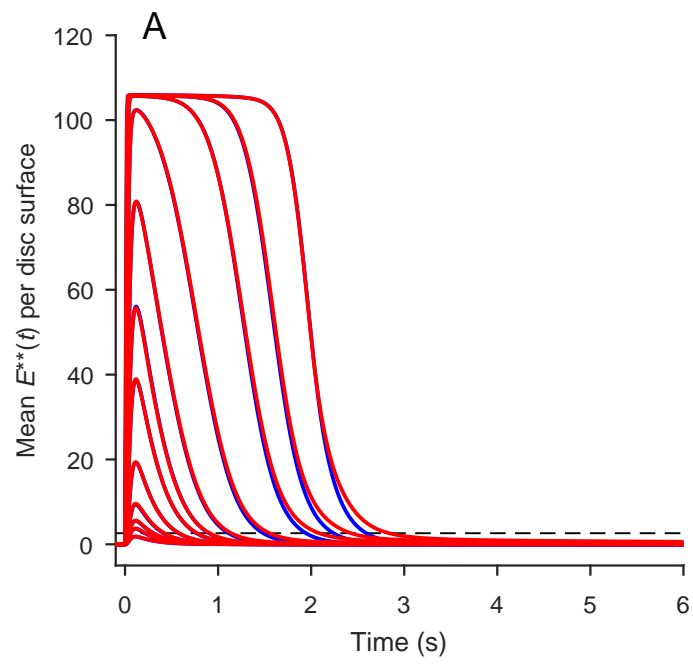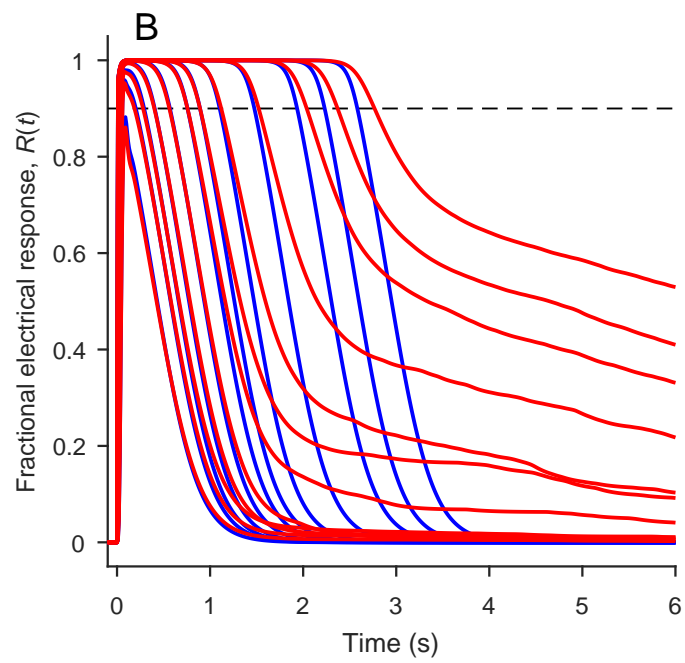

Supplement: Code and simulated data [file rsob190241supp2.zip › Figures/Fig_09_Recovery_Aberrants.pdf]

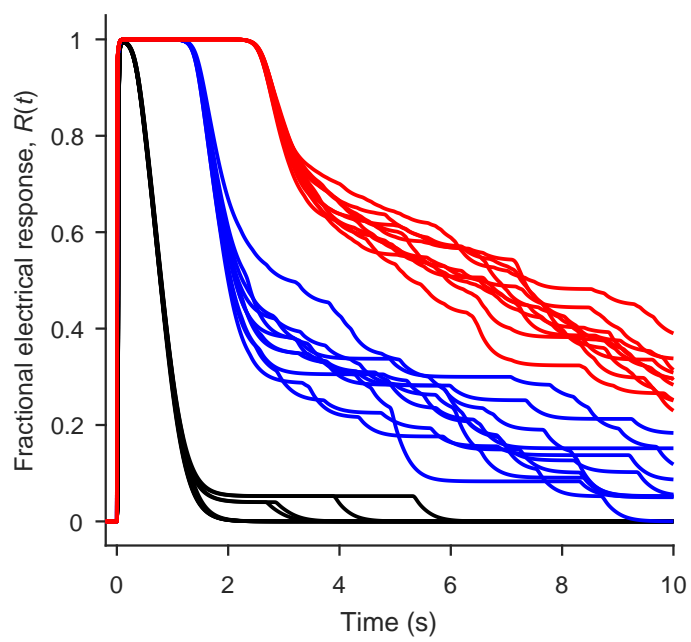

Supplement: Code and simulated data [file rsob190241supp2.zip › Figures/Fig_10_Recovery_Fluctuations.pdf]

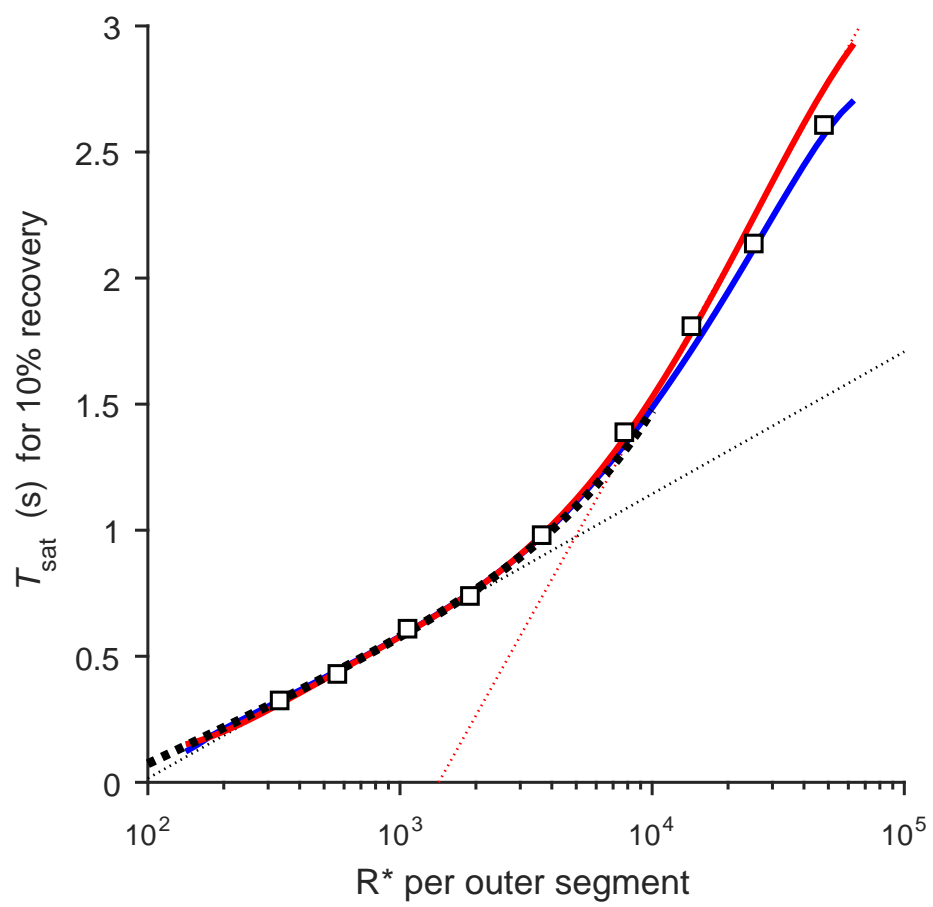

Supplement: Code and simulated data [file rsob190241supp2.zip › Figures/Fig_11_Tsat_v_Phi.pdf]

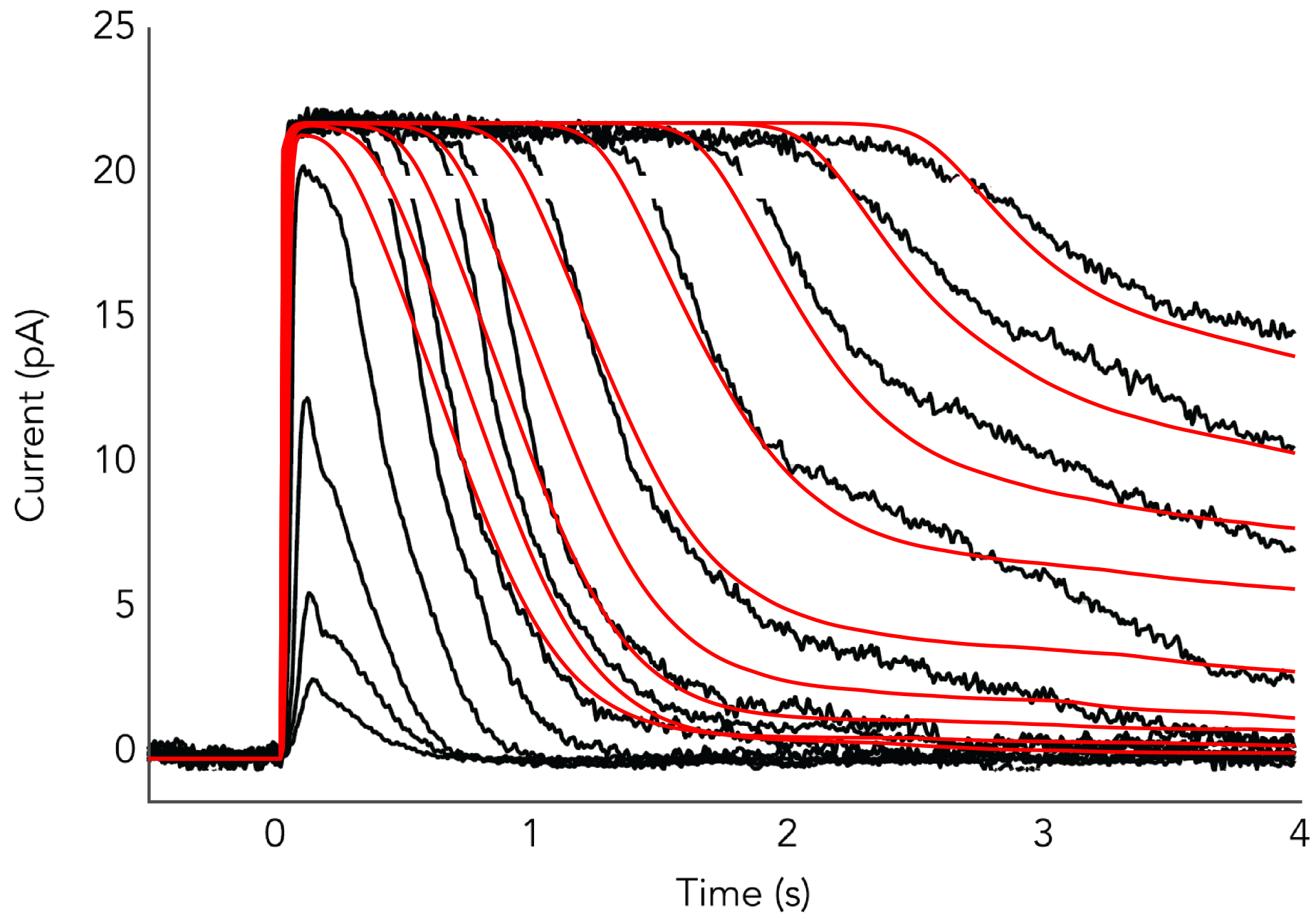

Supplement: Code and simulated data [file rsob190241supp2.zip › Figures/Fig_12_Method_3_Example.pdf]

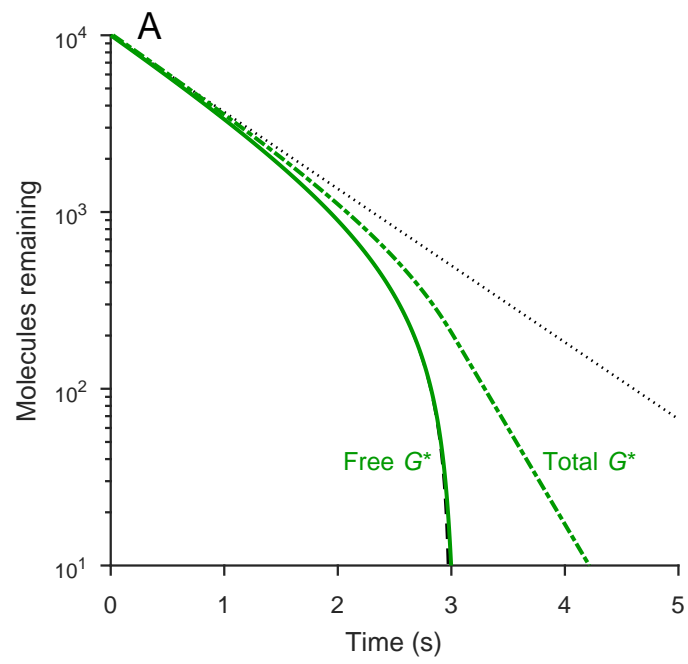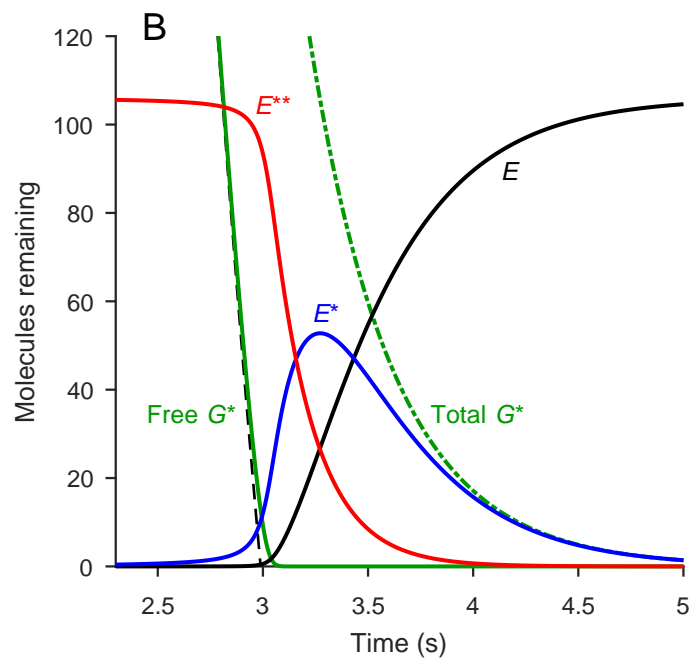

Supplement: Code and simulated data [file rsob190241supp2.zip › Figures/Fig_13_Dimeric_Theory_from_Gs0.pdf]

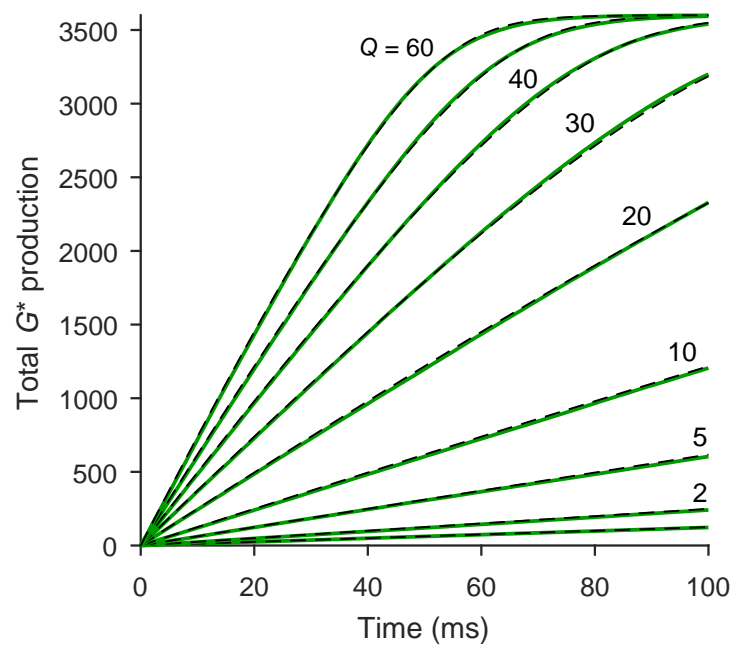

Supplement: Code and simulated data [file rsob190241supp2.zip › Figures/Fig_14_Walk2_G_depletion.pdf]
